# Shared genetic risk factors: implications for treatment of idiopathic pulmonary fibrosis and systemic hypertension

**DOI:** 10.1183/23120541.00457-2023

**Published:** 2023-11-13

**Authors:** Gina Parcesepe, Richard J. Allen, Beatriz Guillen-Guio, Samuel Moss, R. Gisli Jenkins, Louise V. Wain

**Affiliations:** 1 Department of Population Health Sciences, University of Leicester, Leicester, UK; 2 NIHR Leicester Biomedical Research Centre, Leicester, UK; 3 Margaret Turner Warwick Centre for Fibrosing Lung Disease, National Heart and Lung Institute, Imperial College London, London, UK

## Abstract

Idiopathic pulmonary fibrosis (IPF) is the most prevalent progressive idiopathic interstitial pneumonia. It has a poor prognosis (median survival of 2.5–3.5 years from diagnosis) and typically presents in older individuals (mean age 74 years) [1]. Systemic hypertension is highly prevalent and the leading contributor to all-cause death and disability worldwide. Hypertension is a risk factor for common comorbidities of IPF including ischaemic heart disease, and >50% of individuals with IPF have systemic hypertension (defined as ≥130 mmHg systolic blood pressure (SBP) and/or ≥80 mmHg diastolic blood pressure (DBP) [2]). High blood pressure can lead to organ fibrosis or can be a consequence of artery stiffening due to fibrosis [3], and studies have implicated common pathways in both IPF and blood pressure regulation, such as transforming growth factor (TGF)-β signalling and the renin–angiotensin system [4].

## Tweetable abstract

**The discovery of shared genetic associations, with sometimes different directions of effect, has implications for drug target discovery for idiopathic pulmonary fibrosis and systemic hypertension** https://bit.ly/45IqbRv

*To the Editor*:

Idiopathic pulmonary fibrosis (IPF) is the most prevalent progressive idiopathic interstitial pneumonia. It has a poor prognosis (median survival of 2.5–3.5 years from diagnosis) and typically presents in older individuals (mean age 74 years) [1]. Systemic hypertension is highly prevalent and the leading contributor to all-cause death and disability worldwide. Hypertension is a risk factor for common comorbidities of IPF including ischaemic heart disease, and >50% of individuals with IPF have systemic hypertension (defined as ≥130 mmHg systolic blood pressure (SBP) and/or ≥80 mmHg diastolic blood pressure (DBP) [2]). High blood pressure can lead to organ fibrosis or can be a consequence of artery stiffening due to fibrosis [3], and studies have implicated common pathways in both IPF and blood pressure regulation, such as transforming growth factor (TGF)-β signalling and the renin–angiotensin system [4]. Indeed, angiotensin-converting enzyme inhibition, a common treatment for hypertension, has anti-TGF-β and potential antifibrotic effects [5], while rho-associated coiled-coil-containing kinases (ROCK) inhibition, under development for treatment of IPF, is known to affect blood pressure [6]. Given the high co-occurrence and shared pathobiology of these conditions, we hypothesised that there could be shared genetic risk factors for both traits. The observation that genetically supported targets are more likely to be successful in clinical development [7] and the increasing integration of genetics in drug target prioritisation, suggests that understanding the pleiotropic effects of the genes involved could have benefits and implications for drug repurposing and new target discovery for IPF and hypertension.

We utilised the largest available genome-wide association studies (GWAS) of clinically defined IPF [8], and SBP and DBP [9], to examine both genome-wide and locus-specific overlap. Linkage disequilibrium score regression [10] was used to conduct genome-wide correlation. Genome-wide colocalisation was applied using the coloc R package [11] to identify regions with a shared signal at p<10^−5^ between IPF and either SBP or DBP; this was performed by identifying signals at p<10^−5^ in both datasets, and then performing colocalisation. We report signals with a posterior probability of >80% for a shared causal variant (coloc H4) as significant. Signals were mapped to a gene either using prior published evidence or using Open Targets Genetics (OTG). OTG utilises distance from gene, expression quantitative trait loci (QTL), splice QTL and functional annotation to suggest the most likely variant-to-gene mapping.

The IPF GWAS comprised 4125 IPF cases and 20 464 controls. The SBP and DBP GWAS comprised 757 601 general population participants. Both studies included only individuals of genetically determined European ancestry and further included principal components adjustment for fine-scale population structure. There was no genome-wide correlation between IPF and SBP (correlation −0.077, 95% CI −0.142–−0.011; p=0.022) or DBP (correlation −0.027, 95% CI −0.093–0.039; p=0.427). The genome-wide colocalisation analysis identified 18 regions of overlap, of which seven had a posterior probability of a shared causal variant >80% for IPF and blood pressure (table 1). In addition, there was a shared signal at 17q21.31 for which colocalisation could not be reliably performed due to extended linkage disequilibrium across the region. However, the 17q21.31 signals for both IPF and blood pressure tagged an ancestral 1.5 Mb genomic inversion [12] and so this was considered as a shared signal. Of the eight shared signals, three (near *MAD1L1*, *GOLPH3L/HORMAD1* and at 17q21.31) had the same direction of effect on risk of IPF and raised blood pressure (hypertension) and five (near *TERC*, *OBFC1*, *DEPTOR* and at 7q22.1 and 6p21.2) had opposite effects, with the allele associated with increased risk of IPF being associated with decreased blood pressure (and *vice versa* for the other allele) (table 1).

**TABLE 1:** Results of colocalisation analyses, stating the posterior probability that the traits are likely to share a causal variant

**RSID**	**Chromosome**	**Position^#^**	**Coded allele**	**Other allele**	**Coded allele frequency^¶^**	**IPF β (SE)**	**IPF p-value**	**SBP β (SE)**	**SBP p-value**	**H4^+^ SBP (%)**	**DBP β (SE)**	**DBP p-value**	**H4^+^ DBP (%)**	**Gene (source^§^)**
**rs3738485**	1	150552392	G	C	0.45	0.134 (0.027)	9.35×10^−7^	0.164 (0.030)	6.92×10^−8^	90.6^+^	0.018 (0.017)	2.95×10^−1^	75.9	*GOLPH3L/* *HORMAD1* (OTG)
**rs12696304**	3	169481271	G	C	0.28	0.256 (0.030)	8.45×10^−18^	−0.281 (0.035)	4.23×10^−16^	98.9^+^	−0.088 (0.020)	9.94×10^−6^	48.0	*TERC* [8]
**rs1214759**	6	43352980	G	A	0.32	0.165 (0.029)	1.71×10^−8^	−0.333 (0.032)	4.79×10^−25^	99.4^+^	−0.135 (0.019)	2.51×10^−13^	98.6^+^	6p21.2 [8]
**rs12699415**	7	1909479	A	G	0.42	0.236 (0.027)	7.85×10^−18^	0.228 (0.031)	1.82×10^−13^	84.9^+^	0.124 (0.018)	3.30×10^−12^	75.3	*MAD1L1* [8]
**rs2897075**	7	99630342	T	C	0.38	0.263 (0.028)	1.77×10^−21^	−0.156 (0.031)	6.49×10^−7^	99.0^+^	−0.101 (0.018)	1.96×10^−8^	<1.0	7q22.1 [8]
**rs28513081**	8	120934126	G	A	0.43	−0.180 (0.030)	1.22×10^−9^	NA	NA	NA	0.084 (0.018)	1.58×10^−6^	89.6^+^	*DEPTOR*^ƒ^ [8]
**rs7100920**	10	105640978	T	C	0.49	0.170 (0.027)	1.67×10^−10^	−0.204 (0.030)	1.62×10^−11^	92.4^+^	−0.079 (0.017)	5.43×10^−6^	18.7	*OBFC1* [8]
**rs2077551**	17	44214888	C	T	0.19	−0.351 (0.038)	1.92×10^−20^	−0.271 (0.042)	8.75×10^−11^	NA	−0.146 (0.024)	1.24x10^−9^	NA	17q21.31^##^ [8]

RSID: reference single-nucleotide polymorphism cluster identifier; IPF: idiopathic pulmonary fibrosis; SBP: systolic blood pressure; DBP: diastolic blood pressure; OTG: Open Targets Genetics; NA: not applicable. ^#^: build GRCh37; ^¶^: in IPF dataset; ^+^: the posterior probability that the trait is likely to share a causal variant with IPF (probability >80% for a shared causal variant); ^§^: whether the gene information came from variant-to-gene mapping analyses from a prior paper [8] or OTG; OTG utilises distance from gene, expression quantitative trait loci (QTL), splice QTL and functional annotation to suggest the most likely variant-to-gene mapping; ^ƒ^: *DEPTOR* was not in the genome-wide colocalisation for SBP (not present at p<10^−5^ in dataset); ^##^: colocalisation was not performed for 17q21.31 due to extended linkage disequilibrium across the region.

Our study identified shared genetic associations between IPF and blood pressure traits with genetic variants at some loci increasing the risk of both IPF and hypertension, while at other loci, the same genetic variants increased risk of one trait while being protective against the other.

*MAD1L1* (mitotic arrest deficient 1 like 1) encodes a component of the mitotic spindle assembly checkpoint and is a shared and consistent signal of association between IPF and hypertension. Common and rare genetic variants at the *KIF15* (kinesin family member 15) gene [13], and a rare variant in *SPDL1* (spindle apparatus coiled-coil protein 1) [14] have also been associated with pulmonary fibrosis. Collectively, these associations implicating genes with roles in mitotic spindle assembly implicate a reduction in epithelial cell proliferation and increased senescence as a driver of fibrosis. Endothelial cell senescence may also contribute to increased vascular ageing, which promotes hypertension [15]. These data support the development of senolytic therapies that may be effective for both IPF and hypertension [16, 17].

*DEPTOR* (DEP domain-containing mTOR-interacting protein) encodes a mammalian target of rapamycin (mTOR) inhibitor and decreased *DEPTOR* gene expression was associated with increased risk of IPF [18]. Targeting the mTOR pathway has been highlighted as a promising new therapeutic avenue for IPF [19]. Our findings suggest that intervening in this pathway may have potential adverse effects on blood pressure.

There was a significant difference in sample size for the GWAS used in our analyses and this will have affected the statistical power to detect signals in the smaller IPF dataset, meaning that there may be additional shared signals that we have not detected. Our genome-wide approach utilising a more lenient threshold than the commonly used genome-wide threshold of p<5×10^−8^ will have partly mitigated this, but it is likely that more shared signals will be discovered as sample sizes increase. We have used published datasets that undertook adjustment for appropriate demographic covariates and restriction to individuals of European ancestry. Differences in age, sex and smoking history between the datasets may mean that we may have missed additional shared signals in our study. For example, where there may be interaction effects for either trait. None of the shared signals we highlight are associated with smoking.

We only report signals with a single shared causal variant for IPF and blood pressure. It is plausible that distinct causal variants could exert their effect through the same nearby gene or different genes in the same molecular pathway; however, this study was not designed to detect such effects. It is likely that genetic effects may be tissue-specific and that different regulatory pathways may be involved in the expression of the same gene in different tissues.

Our findings provide support that IPF and systemic hypertension have shared mechanisms which may promote both fibrosis and hypertension, as well as others where there are opposite directions of effect. Given the frequency of hypertension and ischaemic heart disease as comorbidities in IPF, and the effect of potential antifibrotics on blood pressure (and *vice versa*), it is important to understand the mechanisms of such shared pathways and to ensure that genetically indicated potential adverse effects are proactively monitored in future clinical trials for new drugs.
